# Convergence of visual and whisker responses in the primary somatosensory thalamus (ventral posterior medial region) of the mouse

**DOI:** 10.1113/JP272791

**Published:** 2016-09-15

**Authors:** Annette E Allen, Christopher A. Procyk, Timothy M. Brown, Robert J. Lucas

**Affiliations:** ^1^ Faculty of Life SciencesUniversity of ManchesterManchesterUK

**Keywords:** multisensory, thalamus, vision, VPM

## Abstract

**Key points:**

- Using *in vivo* electrophysiology, we find that a subset of whisker‐responsive neurons in the ventral posterior medial region (VPM) respond to visual stimuli.
- These light‐responsive neurons in the VPM are particularly sensitive to optic flow.
- Presentation of optic flow stimuli modulates the amplitude of concurrent whisker responses.
- Visual information reaches the VPM via a circuit encompassing the visual cortex.
- These data represent a new example of cross‐modal integration in the primary sensory thalamus.

**Abstract:**

Sensory signals reach the cortex via sense‐specific thalamic nuclei. Here we report that neurons in the primary sensory thalamus of the mouse vibrissal system (the ventral posterior medial region; VPM) can be excited by visual as well as whisker stimuli. Using extracellular electrophysiological recordings from anaesthetized mice we first show that simple light steps can excite a subset of VPM neurons. We then test the ability of the VPM to respond to spatial patterns and show that many units are excited by visual motion in a direction‐selective manner. Coherent movement of multiple objects (an artificial recreation of ‘optic flow’ that would usually occur during head rotations or body movements) best engages this visual motion response. We next show that, when co‐applied with visual stimuli, the magnitude of responses to whisker deflections is highest in the presence of optic flow going in the opposite direction. Importantly, whisker response amplitude is also modulated by presentation of a movie recreating the mouse's visual experience during natural exploratory behaviour. We finally present functional and anatomical data indicating a functional connection (probably multisynaptic) from the primary visual cortex to VPM. These data provide a rare example of multisensory integration occurring at the level of the sensory thalamus, and provide evidence for dynamic regulation of whisker responses according to visual experience.

## Key points

- Using *in vivo* electrophysiology, we find that a subset of whisker‐responsive neurons in the ventral posterior medial region (VPM) respond to visual stimuli.
- These light‐responsive neurons in the VPM are particularly sensitive to optic flow.
- Presentation of optic flow stimuli modulates the amplitude of concurrent whisker responses.
- Visual information reaches the VPM via a circuit encompassing the visual cortex.
- These data represent a new example of cross‐modal integration in the primary sensory thalamus.

**Abbreviations**
cpd: cycles per degree
CSD: current source density
dLGN: dorsal lateral geniculate nucleus
DS: direction selectivity
EYFP: enhanced yellow fluorescent protein
FR: firing rate
LP: lateral posterior thalamic nuclei
POm: posteromedial complex of the thalamus
PSTH: peri‐stimulus time histogram
S1: primary somatosensory cortex
V1: primary visual cortex
VPM: ventral posterior medial region of the thalamus
WGA: wheat germ agglutinin

## Introduction

The senses originate with specialized receptor neurons and are conveyed through sense‐specific thalamic nuclei to areas of the cortex whose activity is dominated by input from one sensory modality (Sherman *et al*. 2006). At some point, however, the brain must integrate information across sensory modalities. Thus, appropriate interpretation of one sensory input can often be aided by contextual information provided by another. Furthermore, merging sensory information is an essential step towards producing a coherent representation of the local environment and our place in it.

There are established examples of multisensory integration at many points in the progression of sensory information, including primary sensory cortices themselves, and secondary cortical and sub‐cortical structures (reviewed by Ghazanfar & Schroeder, 2006; Driver & Noesselt, 2008; Stein & Stanford, 2008; Cappe *et al*. 2012). Such integration is less well established for the primary sensory thalamus. There are reports of interactions between visual and auditory information in the lateral geniculate and medial geniculate nuclei (Komura *et al*. 2005; Noesselt *et al*. 2010), and more widespread vestibular influences throughout the thalamus (reviewed by Wijesinghe *et al*. 2015). Nevertheless, to a first approximation, ascending thalamo‐cortical sensory pathways are generally thought of as being dedicated to the processing of a single sensory modality.

Here we present a new example of sensory integration occurring in a primary sensory thalamic structure: the ventral posterior medial region of the thalamus (VPM). The VPM is the thalamic relay centre for the rodent lemniscal pathway. It receives vibrissal information from the trigeminal complex and transfers it to the barrel cortex. Following early descriptions of somatotopic organization in this region (Waite, 1973; van der Loos, 1976), the VPM and associated barrel cortex have been widely studied as a model of sensory processing. Interactions between visual and vibrissal responses have been described in the cortex (Olcese *et al*. 2013; Sieben *et al*. 2013; Stehberg *et al*. 2014), colliculus (reviewed by Stein & Stanford, 2008) and dorsal striatum (Reig & Silberberg, 2014) but, to our knowledge our data are the first to report such an interaction at the earliest stage of somatosensory processing, the primary thalamus.

## Methods

### Animals

Mice were bred at the University of Manchester and housed under a 12:12 h light/dark cycle, with food and water available *ad libitum*. Recordings were made in 11 male *Opn1mwR* mice, from a C57BL/6; 129sv mixed strain background, and 15 male C57BL/6 mice, aged 3–5 months. Both visual and vibrissal responses were equivalent in these two genotypes.

### Ethical approval

The care and use of all mice in this study was carried out in strict accordance with UK Home Office regulations, UK Animals (Scientific Procedures) Act of 1986 (revised in 2012) and approved by the local Manchester Animal Welfare and Ethical Review Board (AWERB reference 50/02506). *In vivo* recovery surgery was performed under isofluorane anaesthesia. All *in vivo* surgical procedures were performed under terminal urethane anaesthesia. In both cases, all efforts were made to minimize suffering.

### *In vivo* neurophysiology

Mice were anaesthetized with an intraperitoneal injection of urethane (1.7 g kg^−1^; 30%, w/v; Sigma Aldrich, St Louis, MO, USA) and held in a stereotaxic frame (SR‐15M; Narishige International Ltd, London, UK). Pupil dilatation was achieved through application of atropine (Sigma Aldrich) to the stimulated eye. Mineral oil (Sigma Aldrich) was also applied to each eye to retain corneal moisture. Throughout experimentation, core body temperature was maintained at ∼37°C via a homeothermic heat mat (Harvard Apparatus, Edenbridge, UK). The skull was exposed via a midline scalp incision, and a hole drilled in the skull directly above the posterior thalamus (medial–lateral: 1.4 mm; anterior–posterior: −1.8 to 2.1 mm, relative to bregma) according to a stereotaxic mouse atlas (Paxinos & Franklin, 2001). A 32‐channel multi‐microelectrode (NeuroNexus Technologies Inc., Ann Arbor, MI, USA) was lowered ∼3.5 mm into the posterior thalamus. In a subset of recordings, the somatosensory cortex (S1 barrel field) was also targeted (medial–lateral: 1 mm; anterior–posterior: −2.5 to 2.95 mm, relative to bregma), with 32‐channel recording electrode lowered ∼0.75 mm at an angle of 20 deg. The recording electrode consisted of four silicon substrate shanks, 200 μm apart and 5 mm long, with eight iridium electrode sites arranged vertically on each shank (177 or 413 μm^2^ surface area, 50 μm apart; A4×8‐5mm‐50‐200). A Recorder64 recorder system (Plexon, Dallas, TX, USA) was used to acquire signals throughout experimentation. Signals were amplified by a 20× gain AC‐coupled headstage (Plexon) followed by preamplifier conditioning providing a total gain of 3000×. In some cases, a single glass recording electrode was used to record in the VPM. Borosilicate glass micropipettes were pulled to achieve a resistance of 15–20 MΩ, and were filled with 4% Chicago Sky Blue (Sigma Aldrich) in 2 m NaCl. The signal was also recorded using the Plexon Recorder64 system. Data were high‐pass (300 Hz) filtered and time‐stamped neural waveforms were digitized simultaneously from all channels (or a single channel) at a rate of 40 kHz. Local field potential (LFP) data were also acquired by low‐pass filtering the data (1 kHz, first‐order Butterworth), which were digitized continuously at a rate of 10 kHz.

### Histology

To determine the location of recording sites, the multichannel recording electrode was dipped in fluorescent dye (Cell Tracker CM‐DiI; Invitrogen, Carlsbad, CA, USA) prior to insertion. In some recordings, specific electrode sites were also electrolytically lesioned. Following recordings, animals were perfused with 0.9% saline followed by 4% paraformaldehyde in 0.1 m phosphate buffer. The brain was then removed and post‐fixed overnight in 4% paraformaldehyde, and after cryoprotection 99 μm coronal sections were cut using a sledge microtome, mounted onto glass slides and visualized with an Olympus BX51. DiI clearly demarcated the position of electrode shanks in brain sections, allowing the position of electrode sites to be estimated with good precision in the medio‐lateral and rostro‐caudal planes. Position in the dorso‐ventral dimension was estimated by the depth of dye staining and accorded well with lesioning of tissue at known electrode sites, as well as estimates based upon micromanipulator extension that suggest a resolution of ∼50 μm in this dimension. Sections were then aligned with the corresponding mouse atlas sections (Paxinos & Franklin, 2001) to establish the anatomical location of each recording site. In some experiments, the anatomical location of VPM was verified with cytochrome oxidase staining, consistent with Liu *et al*. (1993). To verify the position of the glass recording electrode, at the end of these experiments the recording site was marked by iontophoretical deposition of Chicago Sky Blue (0.3 μA anodal current/16 min).

### Visual stimuli

Mice were left for > 1 h prior to recordings to dark adapt and to allow neuronal activity to stabilize following electrode placement. For full field stimuli, a 460 nm LED (Cairn Research Ltd, Faversham, UK) fitted with a band‐pass filter (half‐peak width = 10 nm) was focused onto a 5 mm diameter circle of opal diffusing glass (Edmund Optics Inc., York, UK) placed ∼5 mm from the eye contralateral to the recording site. A National Instruments card (USB‐6229) controlled by programs written in LabVIEW (version 8, National Instruments, Bethesda, MD, USA) was used to present 30 s stimuli with periods of 300 s darkness between each light pulse (providing 14.9 log photons cm^–2^ s^–1^ respectively at the level of the cornea).

Patterned visual stimuli were presented with a video monitor (34 × 27 cm) positioned 10 cm from the contralateral eye centre (occupying 119.1 deg × 106.9 deg; intensity of black and white = 430 and 3.3 cd m^–2^, respectively). Although stimuli were sometimes visible to the ipsilateral eye, when stimuli were positioned to target the ipsilateral eye directly, we found no evidence of responses in the VPM. Stimuli were generated with customized software written using MATLAB (R2012a; The Mathworks, Natick, MA, USA) running Psychophysics Toolbox extensions (Brainard, 1997). All visual stimuli were adjusted to account for variations in visual angle, so that stimuli presented in the centre or extremes of the monitor occupied equivalent visual angles. Receptive fields were mapped with the presentation of vertical or horizontal bars, which occupied ∼12.5 deg of visual space (250 or 500 ms black bars on a white background, and 250 or 500 ms white bars on a black background). Drifting gratings were presented across the entirety of the screen, with an inversion every 500 ms, at three spatial frequencies: 0.06, 0.13 and 0.24 cycles per degree (cpd). Drifting gratings were presented in eight directions of motion for 10–50 s, either in sequence or in a randomized order of presentation. Isolated presentations of drifting gratings were also used in a subset of experiments, transitioning from grey screen to 2 s drifting grating stimulus moving naso‐temporally (0.24 cpd). More complex visual stimuli consisting of either coordinated or uncoordinated movement of 16/32 evenly sized white squares presented on a black background were also presented. These squares occupied ∼12.5 × 12.5 deg of visual space, and moved at a speed of 20 deg s^–1^ in coordinated or opposing directions. Responses to a naturalistic stimulus, occupying 115 × 100 deg of visual space, were also recorded. Thirty repeats of a 33 s epoch of a movie recorded from the viewpoint of a mouse during natural exploratory behaviour (Froudarakis *et al*. 2014) were presented in greyscale, with concurrent 0.75 Hz targeted whisker movement (see below). All light measurements were performed using a calibrated spectroradiometer (Bentham Instruments, Reading, UK).

### Whisker stimuli

Responses were also recorded to targeted movement of the contralateral whiskers using a 10 ms air‐puff, presented at 1, 2 or 10 Hz. Air puffs were provided using a timed valve connected to narrow (<2 mm diameter) tubing; this was placed 1 cm from the whiskers, and directed either naso‐temporally or dorso‐ventrally at the whisker ends (to avoid any stimulation of skin receptors). This stimulus moved an unbiased population of whiskers, and was approximately ∼20 deg for 1 Hz, 10 ms stimulus. The frequency of stimulation was controlled with a National Instruments card (USB‐6229) controlled by programs written in LabVIEW (version 8, National Instruments). Responsive units were those which showed discernible peaks in peri‐stimulus time histograms (PSTHs), exceeding the 99% confidence limits that were calculated from a Poisson distribution based on pre‐stimulus spike firing. Wherever possible, to ensure the origin of responses was with the movement of whiskers (and not owing to movement of fine hairs or the sound of an air‐puff), at the end of a recording session whiskers were cut and air‐puff stimuli were repeated – in these cases, whisker responses were abolished (see Fig. 2).

### Muscimol application

In four mice the GABA_A_ receptor agonist muscimol (Sigma Aldrich) was used to silence activity in the visual cortex. In these experiments, a second craniotomy was made above the visual cortex (contralateral to visual and whisker stimuli; coordinates from bregma: anterior–posterior: −4.5 mm, medial–lateral: −2.5 mm), and a second 4 × 8 recording electrode was inserted at an angle of 40 deg to a depth of ∼ 600 μm. Following an initial presentation of recordings, 100 μl of 1 mm muscimol (diluted in 0.9% saline) was applied to the exposed cortex, and light‐evoked responses were tracked for the following 20 min. After this time, and the cessation of light‐evoked responses in the cortex, activity in the VPM was again recorded in response to full field light steps, drifting gratings and whisker stimuli.

### Electrical stimulation

In two mice, a craniotomy was made above the visual cortex (coordinates as previous), and a 1 × 32 recording/stimulating electrode was inserted at an angle of 40 deg to a depth of ∼ 600 μm. A second 4 × 8 recording electrode was inserted into the VPM (coordinates as previous). Alternating sites on the V1 1 × 32 electrode were used for recording and stimulation. Pairs of stimulation sites were chosen based on the location of light‐evoked responses recorded in V1, and biphasic square‐wave 100 μs, 80 μA current pulses were passed between these pairs (PlexStim 2.0, Plexon). Stimulation‐evoked responses were then recorded in the VPM, and analysed as described below.

### Virus and tracer injections

Five Ai32 mice [homozygous for the Rosa‐CAG‐LSL‐ChR2(H134R)‐EYFP‐WPRE conditional allele] were anaesthetized with 1–2% isofluorane in oxygen. The skull was exposed via midline scalp incision, and a hole drilled in the skull directly above VPM (medial–lateral 1.6 mm; anterior–posterior: −2.0 mm; relative to bregma) according to a stereotaxic mouse atlas (Paxinos & Franklin, 2001). A virus [AAV‐EF1a‐mCherry‐IRES‐WGA‐Cre (Gradinaru *et al*. 2010)] was injected using a Nanoject II (Drummond, Broomall, PA, USA) through a glass pipette. Several injections of 69 nl each were made at depths of −3.8 to −3.5 mm. Mice were given bupremorphine as an analgesic following surgery. Four weeks after injection, two mice were perfused with 0.9% saline followed by 4% paraformaldehyde in 0.1 m phosphate buffer, and three were used for electrophysiology (see below) prior to perfusion fixation.

### Optogenetic stimulation

In three mice that had previously been injected with a viral tracer (see above), to activate cells expressing ChannelRhodopsin in the visual cortex, we made a craniotomy over the visual cortex (coordinates as previous), above which an optic fibre (200 μm diameter) was positioned, connected to a DPSSL laser (λ_max_ = 470 nm; AixIZ LLC, London, UK) with an operating power of 60 mW. In these experiments, the craniotomy drilled for viral injection was reopened, and a 4 × 8 recording electrode was inserted to a depth of ∼ 3.5 mm. Laser‐evoked responses were then recorded in the VPM, and analysed as described below.

### Immunohistochemistry

Paraformaldehyde‐fixed brains were sectioned at 50 μm thickness using a sledge microtome. Fluorescence immunohistochemistry was performed as described previously (Allen *et al*. 2011), and was used to highlight mCherry and enhanced yellow fluorescent protein (EYFP) expression. In brief, primary antibodies used in these studies were rabbit anti‐dsRed (Clontech 632496; 1:500; Palo Alto, CA, USA) and chicken anti‐GFP (Abcam ab13970; 1:1000; Cambridge, MA, USA), diluted in 4% normal donkey serum (Sigma Aldrich, Poole, UK) in 0.1 m phosphate buffer. The secondary antibodies were Alexa 488 conjugated donkey anti‐chicken (Jackson Immunoresearch, West Grove, PA, USA) and Alexa 546 conjugated donkey anti‐rabbit (Life Technologies) at 1:200, diluted in 0.1 m phosphate buffer. Images were collected on a Leica TCS SP5 AOBS inverted confocal microscope (detection mirror settings; FITC 494–530 nm; red 570–665 nm; using the 488 and 561 nm laser lines respectively), or on a Leica DM2500 microscope [with a CoolLED pE300 LED light source filtered through a Chroma L5 ET(k) or Chroma Y3 ET(k) filter set] using a Leica DFC365 FX camera. To eliminate any potential cross‐talk between channels, the images were collected sequentially. When acquiring 3D optical stacks the confocal software was used to determine the optimal number of Z sections. Only the maximum intensity projections of these three‐dimensional stacks are shown in the results.

### Analysis of electrophysiological data

Neural waveforms were processed using Offline Sorter (version 2.8.8; Plexon). Cross‐channel artifacts were identified and removed, and single‐unit spikes were detected and categorized based on the spike waveform via a principal component analysis, whereby distinct clusters of spikes were readily identifiable that showed a clear refractory period in their interspike interval distribution. In addition, isolation distances (Harris *et al*. 2001) were quantified for all isolated units and a threshold of an isolation distance > 50 was exceeded in all but six isolated units. Single‐unit data were then further examined using NeuroExplorer (version 4.032; Nex Technologies, Reston, VA, USA), where PSTHs were generated to evaluate changes in firing rate. Light/whisker/electrical/ChannelRhodopsin responsive units were identified as those that showed discernible peaks in peri‐stimulus time histograms, which exceeded the 99% confidence limits, calculated from a Poisson distribution based on pre‐stimulus spike firing. Response latencies were then calculated as the time to half maximum response, based on mean‐evoked firing rates. Durations were quantified as the time over which responses were maintained above 99% confidence limits. All data were visualized and statistically examined using Office Excel (2003; Microsoft Corporation), GraphPad (version 6; GraphPad Software, Inc., La Jolla, CA, USA) and custom‐made programs in MATLAB (R2012a, The Mathworks).

#### Receptive fields

To assess the receptive field size of cells, PSTHs were plotted of bars positioned at various points in visual space at horizontal and vertical orientations. These responses were used to reconstruct 2D receptive fields of units found in the VPM. The response from any particular bar position was classed as significant if it exceeded 2× standard deviations of the pre‐stimulus firing rate. Evoked responses could not be easily fitted with Gaussian curves as they covered large regions of visual space. Instead, the unbroken area over which significant responses were evoked was instead used as a measure of receptive field size.

#### Drifting gratings

The responses to drifting gratings were examined with power‐spectral analysis. We examined whether a peak was present at the 2 Hz inversion frequency of the drifting stimulus, which was tested against a control condition of the pre‐stimulus power spectrum. To establish whether cells showed any direction selectivity, the mean firing rate throughout the presentation of drifting gratings in each orientation was calculated, and the response at the preferred direction (*D*_pref_; where the maximum firing rate occurred) was compared with movement in the opposite direction (*D*_null_; null direction) to generate the DS index with the following equation: (*D*_pref_ ‐ *D*_null_)/(*D*_pref_ + *D*_null_) (Zhao *et al*. 2013). Any unit with a DS index > 0.33 was classed as direction sensitive. A Watson–Williams multi‐sample test for equal means was used to compare the direction tuning preference or response amplitudes between groups of neurons, using a MATLAB toolbox of Circular Statistics (Berens 2009).

#### Current source density

Current source density (CSD) was generated from light‐evoked, averaged LFPs by taking the second‐order spatial derivative across the electrode sites and interpolated to produce a smoothed CSD map (for detailed methods see Davis *et al*. 2014).

#### Cross‐correlation analyses

Cross‐correlations were calculated between pairs of neurons recorded within V1 and VPM concurrently. Spontaneous firing rates of pairs of neurons were correlated with a shifting time window of 1 ms. Significant correlations were those that exceeded the 2× standard deviations of the baseline correlation. Shuffling the firing rate of V1 neurons and repeating the same analyses resulted in no pairs of neurons crossing this significance threshold.

#### Natural movies

Responses to whisker stimuli during presentations of the video were analysed in two ways. Pearson's correlation coefficients were calculated for firing activity (PSTHs) across 30 repeats of the movie. The PSTH during each repeat of the movie was then shuffled, whereby localized PSTHs were still aligned to whisker stimuli but randomized across movie phase, and correlation coefficients were recalculated.

## Results

### Visual responses in the VPM

**Figure 1:**
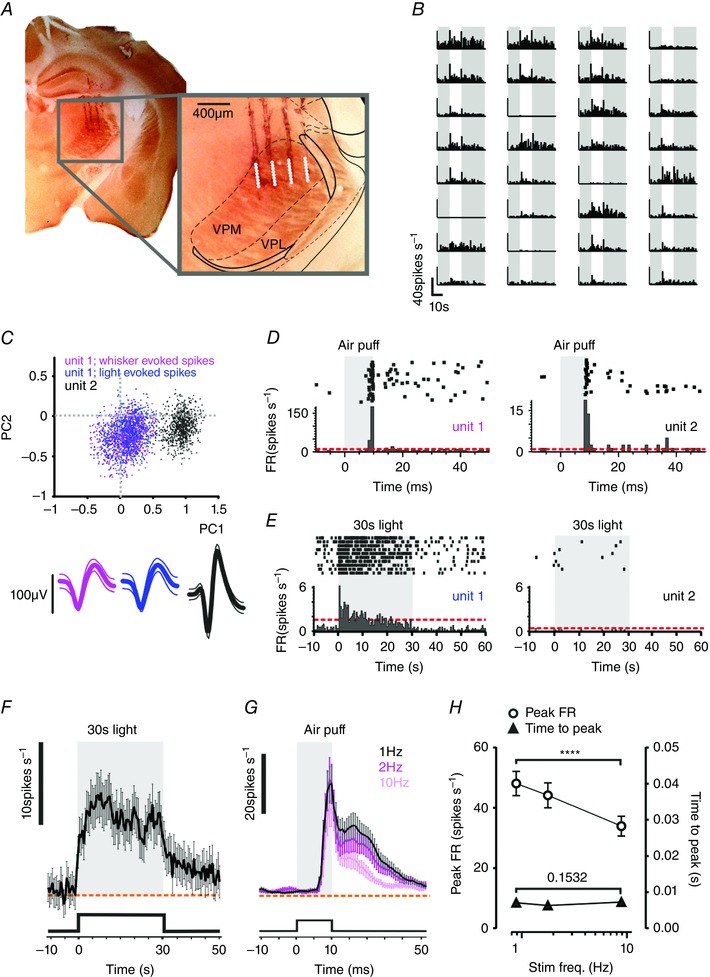
Visual responses in the mouse VPM *A* and *B*, a representative placement of our 4 × 8 multichannel recording probe in the mouse VPM (*A*) and associated light responses (*B*). *A*, half coronal section in which tracks of the four‐shank electrode are visible owing to tissue damage and deposition of DiI applied to the probe prior to insertion. Inset shows expanded view of recording site with approximate boundaries of major brain nuclei. Location of the VPM was confirmed with cytochrome oxidase staining (brown). *B*, multi‐unit, light‐evoked changes in firing rate at each of 32 recording sites for this placement. Responses are mean change in firing rate to 10 presentations of a full field 10 s stimulus. Grey bars indicate periods of darkness before and after visual stimulus. *C*, separation of two representative single units by principal component analysis. Scatter plot shows first two principal components (PC1 and PC2) of two separated units; waveforms underneath show spikes from unit 1 elicited by whisker (pink) and light (blue) stimuli and from the separated unit 2 (black). *D* and *E*, peri‐event raster (above) and histograms for units 1 and 2 from *C* over multiple presentations of whisker (*D*, 10 ms air‐puff moving contralateral whiskers) or light (*E*, 30 s contralateral full field stimulus; 14.9 log photons cm^–2^ s^–1^) stimuli. Grey shading indicates stimulus presentation. *F*, mean ± SEM firing rate of all neurons classed as light responsive (77/231 units recorded in seven mice) in response to 30 s contralateral full field stimulus. Orange dashed line shows baseline firing rate. Grey shading indicates stimulus presentation. *G*, mean ± SEM firing rate of VPM neurons in response to 10 ms air puff at 1, 2 and 10 Hz (black, pink and pale pink, respectively; *n = *46 units). Grey shading indicates stimulus presentation. *H*, quantification of response amplitude and latency of whisker responses in light responsive units at three tested frequencies; RM one‐way ANOVAs of these parameters revealed significant differences in amplitude (circles; *P < *0.0001) but not latency (triangles; *P = *0.15). Data show mean ± SEM changes in firing rate (*n = *46). [Colour figure can be viewed at wileyonlinelibrary.com]

While characterizing the visual response properties of the mouse thalamus [targeting regions known to receive visual input: dorsal lateral geniculate nucleus (dLGN), posteromedial complex of the thalamus (POm) and lateral posterior thalamic nuclei (LP)], we serendipitously also recorded light responses in regions outside of these centres. *Post hoc* histological reconstruction of our recording sites indicated that these light responses originated in a ventro‐medial region identified as the VPM, based upon comparisons with anatomical landmarks in the appropriate atlas in the coronal plane (Paxinos & Franklin, 2001). The VPM is a critical relay for vibrissal signals that has not previously been reported to respond to visual stimuli, and can be distinguished from neighbouring parts of the thalamus using a cytochrome oxidase counter‐stain. In a series of follow‐up recordings we therefore specifically targeted the VPM based upon its stereotaxic coordinates and reconstructed recording sites in coronal brain sections counter‐stained with cytochrome oxidase (Fig. 1 *A*). Using 32 channel multi‐electrode probes, we isolated 231 single units from seven mice that were located in the VPM (based upon these criteria; Fig. 1 *A–C*). In total, 77 of these neurons responded to a simple 30 s full‐field light pulse (14.9 log photons cm^–2^ s^–1^) with sustained increase in firing, often lasting for the duration of a light exposure [Fig. 1 *E*, *F*; median latency: 0.16 s; increase in firing rate (FR): 12.05 ± 0.85 spikes s^–1^; duration 19.47 ± 1.05 s; mean ± SEM]. These neurons were not localized to any particular anatomical subsection of the VPM.

**Figure 2:**
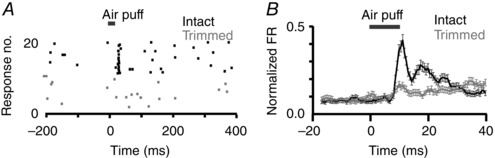
Clipping whiskers abolished responses to air‐puff stimuli *A*, raster plot of a representative unit to 10 ms whisker stimulus with intact (black) and trimmed (grey) whiskers. *B*, mean ± SEM normalized firing rate of all whisker responsive neurons (*n = *144) before (black) and after (grey) whisker trimming. Grey bars indicate air‐puff stimulus.

**Figure 3:**
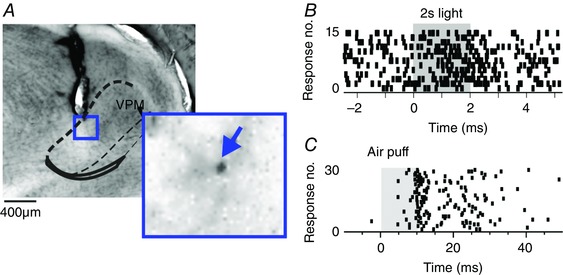
Single channel isolation of cross‐modal responses in VPM *A*, histological section (approximate boundaries of VPM indicated with dashed lines), in which the tract of a single channel, high‐impedance glass electrode probe is visible. Inset shows magnification of cellular iontophoretical deposition of Chicago Sky Blue, highlighted with blue arrow. *B* and *C*, raster plots of a representative single unit isolated with high‐impedance electrode in response to light [*B*; 15 presentations of a full field 2 s stimulus to contralateral eye (grey shading indicates light presentation) and whisker stimuli (*C*; 30 presentations of a 10 ms air puff, moving contralateral whiskers (grey shading indicates air‐puff presentation)]. [Colour figure can be viewed at wileyonlinelibrary.com]

Neurons in the VPM are primarily sensitive to whisker deflections. We therefore next investigated whether light responsive units were also responsive to contralateral whisker movement induced by a simple air‐puff. Indeed, 78% of light‐responsive units showed significant modulations in firing associated with this whisker stimulus (Fig. 1 *D*, *G*). Responses to the air‐puff stimulus were abolished following trimming of whiskers, confirming they indeed arose from movement of the whiskers themselves (Fig. 2). Whisker responses are also found in the neighbouring POm, a location in which we have previously reported light responses (Allen *et al*. 2016) and a known site for multisensory integration (Noseda *et al*. 2010). To confirm our anatomical data indicating that our recordings in this instance were from the more ventro‐lateral VPM, we described the frequency response characteristics for the whisker stimulus. We found that light+whisker responsive units showed significant decreases in amplitude [repeated‐measures (RM) one‐way ANOVA, *P* < 0.0001] but no concomitant change in latency as stimulus frequency increased (RM one‐way ANOVA, *P = *0.1532; Fig. 1 *G*, *H*). This behaviour is consistent with reports from the VPM, but not POm (Diamond *et al*. 1992; Ahissar *et al*. 2000; Bale & Petersen, 2009). As a final confirmation that visual responses are present in the VPM, we used high impedance, single channel glass electrodes in which the recording site could be marked by iontophoretic deposition of Chicago Sky Blue (Fig. 3).

### VPM neurons respond to optic flow

**Figure 4:**
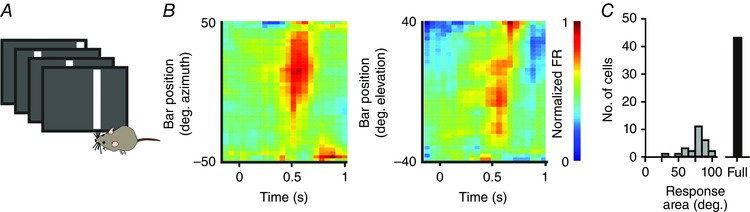
Spatial receptive fields of VPM light responses *A*, cartoon showing receptive field mapping protocol, comprising presentation of bars (500 ms duration, each bar occupying 12.5 deg of visual space) at changing spatial locations. *B*, heat maps for a representative neuron showing the mean normalized firing rate over time (*x*‐axis) to the presentation of bars (as depicted in *A*) at various positions in space (*y*‐axis). Left panel shows responses to vertical bars and right panel to horizontal bars. *C*, histogram showing the distribution of neurons (*n = *69) responding over particular areas of visual space, plus the population of neurons that responded only to full field stimulation. [Colour figure can be viewed at wileyonlinelibrary.com]

Neurons throughout the visual projection respond to full field light pulses of the kind described so far in this study. Nonetheless, their true function is generally to convey and process more complex visual features (Niell, 2015). Our next step was to therefore see how VPM neurons responded to spatially structured stimuli. We began by presenting simple bars in vertical and horizontal orientations [Fig. 4 *A*; an approach we use routinely to map spatial receptive fields in the visual thalamus (Allen *et al*. 2016)]. Surprisingly, 43 out of 69 cells did not show a significant response to these smaller stimuli (see Methods). Of those that did, 43% responded to stimuli across wide regions of visual space (> 50 deg; Fig. 4 *B*, *C*), revealing spatial receptive fields that are much larger than found in conventional visual centres.

**Figure 5:**
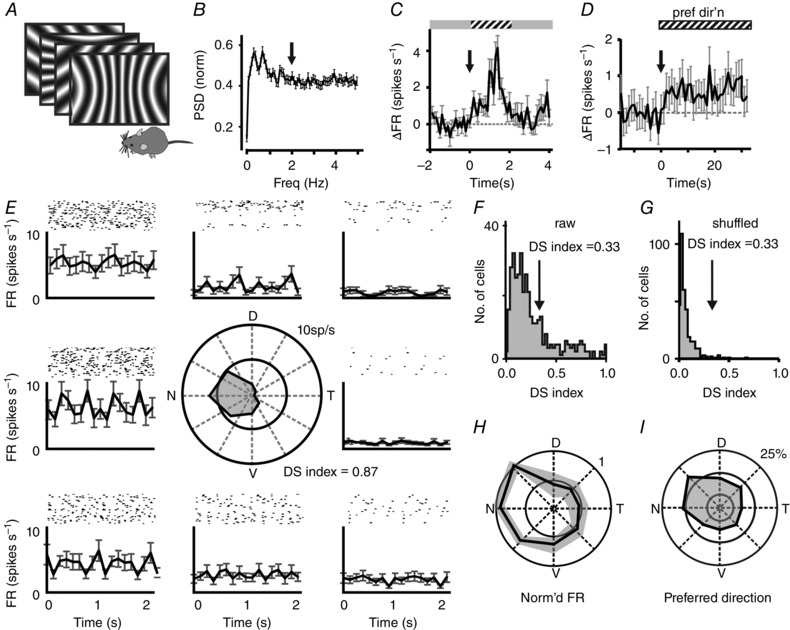
Units in the VPM respond to visual motion *A*, cartoon depicting presentation of drifting grating stimuli, which were presented drifting in eight directions [0.24 cycles per degree (cpd); inversion every 500 ms]. Gratings were adjusted to account for variations in visual angle, so that stimuli presented in the centre or extremes of the monitor occupied an equivalent area of visual space. *B*, mean ± SEM normalized power spectral density (PSD) of 160 light‐responsive neurons. Power spectral densities were computed for all light‐responsive neurons following onset of drifting gratings; no neuron showed a statistical change in power at 2 Hz (arrow) during presentation of drifting grating. *C*, mean ± SEM firing rate of a subset of neurons (*n = *28/160) in response to a transition from grey screen to drifting gratings (2 s presentation of 0.24 cpd grating). Data show baseline‐subtracted change in firing rate for drift responsive neurons. Transition from grey to grating stimulus is depicted above, and with arrow. *D*, mean ± SEM change in firing rate of all DS cells (DS index > 0.33) upon switching to their preferred direction of movement (*n = *107) from a (random) alternative direction of motion. Data show baseline‐subtracted change in firing rate. Transition from gratings of one direction to another is depicted above and with arrow. *E*, the firing rate of an example unit to visual gratings drifting in eight directions (positioned clockwise relative to the direction of motion). For each direction, a double plot of the mean ± SEM firing rate is shown in the lower panel, with raster plots of each repeat shown above. Polar plot in centre shows mean firing rate during presentation of drifting gratings in each direction. D = dorsal, V = ventral, T = temporal, N = nasal. This particular neuron has a high DS index of 0.87. *F* and *G*, the direction selective index for all units is plotted in *F* as a histogram (data shows DS index at 0.24 cpd for all units isolated from VPM; *n = *368). A threshold of > 0.33 was used to categorize cells as direction selective (DS), indicated with arrow. A similar histogram produced for a shuffled version of the data is clustered at low DS indices (*G*). *H*, polar plot showing the normalized firing rate (mean ± SEM for four presentations) from a representative cell showing that the pattern of firing for different directions was retained across repeated presentations. D = dorsal, V = ventral, T = temporal, N = nasal. *I*, polar plot showing the distribution of preferred direction of motion at 0.24 cpd for all DS neurons (*n = *95). D = dorsal, V = ventral, T = temporal, N = nasal.

Given the very poor spatial resolution of VPM light responses, we next considered the possibility that they might respond to a higher‐level abstraction of the visual scene. Visual motion is encoded at the level of retinal ganglion cells (as well as at other points in the visual projection; Niell & Stryker, 2008), and is used as an indicator of self‐motion for systems controlling head and eye movements. Given that other measures of self‐motion (e.g. vestibular; running speed) are known to influence activity in the VPM and other primary thalamic nuclei, we next asked whether VPM neurons were responsive to visual motion, by recording activity during presentation of drifting sinusoidal gratings (Fig. 5 *A*). Consistent with the lack of resolvable spatial receptive fields in VPM neurons, we did not find any VPM unit that showed a modulation in firing that was phase‐locked with the grating (Fig. 5 *B*; see Methods for power spectrum analysis). However, we found that a transition from an isoluminant grey screen to drifting gratings drove significant increases in tonic firing rate in 43% of light responsive neurons (Fig. 5 *C*). In a follow up set of experiments, we found that the effect of drifting gratings on VPM neuron firing rate was particularly dependent upon the direction of motion, with 95 of 368 VPM units having a DS index > 0.33 (see Methods) indicating that they had a ‘preferred’ direction that maximally increased firing (Fig. 5 *D–G*). This direction selectivity was stable over time, with neurons showing consistent responses over repeated exposure to their preferred direction of movement (Fig. 5 *H*). Across the population, there were examples of neurons with direction selectivity for each of the axes tested (Fig. 5 *I*).

**Figure 6:**
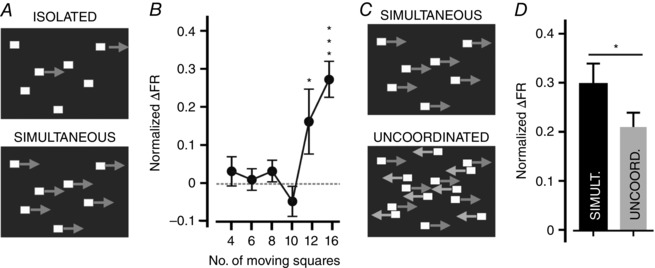
The VPM response to coordinated visual motion *A*, schematics of the stimuli used to test responses to coordinated visual motion. ‘Isolated’ motion (top panel) was created by varying the numbers of these squares remaining static (between four and 12 squares). ‘Simulataneous’ motion (lower panel) was created by coordinated horizontal movement of 16 white evenly sized squares (12.5 × 12.5 deg of visual space) on a black background. *B*, mean ± SEM normalized change in firing rate (relative to baseline) of light‐responsive neurons (*n = *78) during isolated motion of between four and 16 squares. Amplitudes were compared with a one‐way ANOVA with a Dunnett's *post hoc* test comparing each condition with that of no movement; only when 12 or all 16 squares were moving was a significant increase in firing rate induced (^*^ *P < *0.05; ^***^ *P < *0.001). *C*, ‘uncoordinated’ motion (lower panel) was generated by introducing another 16 white squares moving horizontally in the opposite direction. *D*, mean ± SEM normalized change in firing rate (relative to baseline) during presentation of simultaneous or uncoordinated motion (compared with paired two‐tailed *t* test; *P = *0.03, *n = *18).

Full field drifting gratings provide an example of coherent motion across the scene (‘optic flow’), of the sort associated primarily with self‐motion. However, it does not follow that this is the critical feature for eliciting responses in the VPM. We therefore undertook further experiments to explicitly test the hypothesis that these units were responsive to coherent visual motion. We first examined the amount of visual motion that was required to elicit these responses. To this end, we presented a scene with 16 equal sized squares and asked simply how many of them needed to move in order to modulate the firing of VPM neurons. We found that changes in VPM activity occurred only when all or nearly all (at least 12 out of 16) of the blocks moved simultaneously (Fig. 6 *A*, *B*; firing rate compared with RM one‐way ANOVA with Dunnett's *post hoc* test; *P* < 0.05 and *P* < 0.001 for movement of 12 and 16 squares compared with no movement; *P* > 0.05 for all other conditions). We then investigated whether the motion of such objects needed to be in the same direction. We first presented a group of 16 squares moving together in a temporo‐nasal direction. We then added a similar number of squares of the same size and moving at the same speed but in the opposite direction. Neuronal firing was increased compared to baseline (during no visual motion) by this stimulus (paired two‐tailed *t* test comparing change in firing rate from baseline compared for simultaneous and un‐coordinated movement; *P = *0.03) indicating that VPM neurons are excited by even this less coordinated motion. Importantly, however, despite increasing the total amount of movement in the scene we found that the inclusion of additional squares going in the opposite direction suppressed the excitatory effect of the temporo‐nasal drift (Fig. 6 *C*, *D*). It seems then that VPM neurons are generally excited by visual motion, but most responsive to coherent motion of multiple objects across the visual scene.

### Interactions between visual and whisker responses

**Figure 7:**
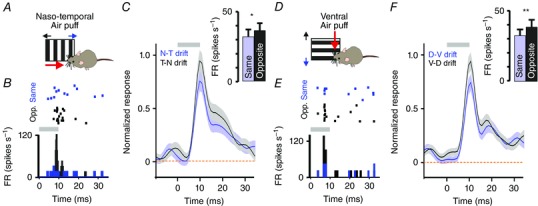
Visual motion modulates the amplitude of whisker responses *A*, cartoon depicting stimulation of whiskers (naso‐temporal whisker deflection; red arrow) with co‐application of naso‐temporal or temporo‐nasal drifting gratings (blue and black arrows, respectively). *B*, raster plot (upper panel) and PSTH (lower panel) of responses of a representative unit to naso‐temporal whisker deflections (10 ms 1 Hz air puff stimulus at time 0) during co‐applied drifting gratings moving in the same (naso‐temporal; blue rasters/PSTH) or opposite (temporo‐nasal; black rasters/PSTH) direction. Grey bar indicates presentation of air‐puff. *C*, mean ± SEM PSTHs of normalized firing rate for units responding to naso‐temporal whisker deflections (10 ms 1 Hz air puff stimulus at time 0) recorded during co‐application of a visual grating moving in the same (blue) or opposite (black) direction. Inset: mean ± SEM peak change in firing rate when gratings were co‐applied in the same or opposite direction (paired two‐tailed *t* test: ^*^ *P = *0.04). Grey bar indicates presentation of air‐puff. *D–F*, as *A–C* for units that responded to ventral whisker deflections (*n = *99) and exposed to ventral (same) or dorsal (opposite) drifting gratings. The amplitudes of whisker responses (*F*, inset) were significantly different in the presence of visual gratings drifting in the same *vs*. opposite direction (paired two‐tailed *t* test: ^**^ *P = *0.0073). [Colour figure can be viewed at wileyonlinelibrary.com]

An obvious next step in understanding the role of light‐evoked responses in the VPM is to establish what impact (if any) visual input has on the VPM's primary function: the transmission and processing of vibrissal information. To examine the impact of cross‐modal responses, we first presented a naso‐temporal whisker deflection and investigated whether the VPM response to this stimulus was modified by concurrent presentation of drifting visual gratings in the same *versus* opposite direction. We found that whisker responses were significantly larger for gratings moving in the opposite (temporo‐nasal) compared to the same (naso‐temporal) direction (Fig. 7 *A–C*; paired two‐tailed *t* test, *P* < 0.05). Rotating our air puff stimulus 90 deg allowed us to make the same comparison for neurons responding to ventral whisker deflections (Fig. 7 *D–F*). Again, responses were significantly larger when visual gratings were presented in the opposite (dorsal) compared to the same (ventral) direction of movement (paired two‐tailed *t* test for ventral whisker deflections, *P* < 0.01; paired two‐tailed *t* test for combined naso‐temporal and ventral whisker deflections with same *vs*. opposite visual motion: *P = *0.0012).

**Figure 8:**
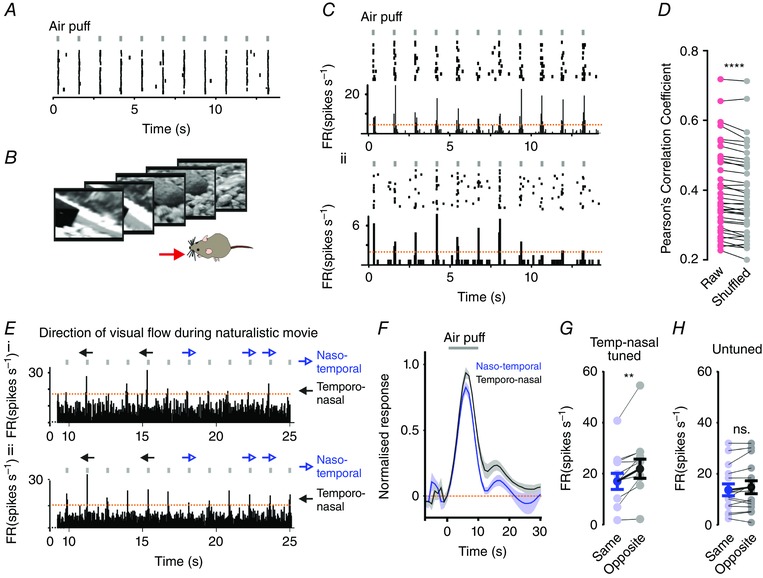
Whisker responses are tuned by a naturalistic representation of visual motion *A*, a representative VPM unit showing highly reproducible responses to repeated whisker stimulations when presented in the dark (raster plot of spikes over 30 repeats of a 15 s trail of air‐puffs). Timing of air puffs is indicated above rasters with grey bars. *B*, example frames taken from a 33 s movie that was presented during co‐application of a whisker stimulus. *C*, responses of two representative units shown as rasters (upper panels) and mean PSTHs (lower panels) to whisker stimuli (indicated by black bars above raster plots) during co‐presentation of a 33 s naturalistic movie (responses shown to 15 repeats of movie). Timing of air puffs is indicated above rasters with grey bars. *D*, aligned scatter plot of Pearson's correlation coefficient calculated for activity across 30 repeats of the movie (red circles) and for shuffled versions of the data (grey circles; activity still aligned to whisker stimulus but shuffled across movie phase) in 39 whisker/light‐responsive units (paired two‐tailed *t* test: *P < *0.0001). *E*, portions of the naturalistic movie that contained epochs of coherent visual motion are indicated above the PSTHs from two representative VPM units, which were tuned to temporo‐nasal visual motion (as determined by responses to drifting gratings). Black arrows indicate motion in preferred (temporo‐nasal) direction; blue arrows in anti‐preferred (naso‐temporal) direction. Timing of air puffs is indicated above rasters with grey bars. Orange dashed lines show confidence interval based on 2× standard deviations of baseline firing rate. *F*, mean ± SEM normalized firing rate in response to whisker stimulation during epochs of the naturalistic movie containing preferred or anti‐preferred motion. Data show responses of direction‐selective neurons tuned to temporo‐nasal motion (*n = *10). Grey bar indicates presentation of air‐puff. *G* and *H*, mean ± SEM change in firing rate in response to whisker stimuli for direction‐selective neurons tuned to temporo‐nasal motion (*G*; *n = *10) or untuned neurons (*H*; *n = *15) during epochs of the naturalistic movie containing temporo‐nasal (black) or naso‐temporal (blue) motion. Neurons tuned to temporo‐nasal motion showed significant differences in amplitude to naso‐temporal whisker deflections during visual motion occurring in the opposite *vs*. same direction (paired two‐tailed *t* test ^**^ *P < *0.01); untuned neurons showed no significant difference in response amplitude (paired two‐tailed *t* test, *P* > 0.05). [Colour figure can be viewed at wileyonlinelibrary.com]

We next tested whether whisker responses in the VPM were modulated by a naturalistic representation of optic flow. To this end, we applied a simple whisker stimulus (1 ms naso‐temporal air puff at 0.75 Hz; Fig. 8 *A*) while presenting a movie of the visual scene as experienced by a mouse during natural exploratory behaviour (Fig. 8 *B*; Froudarakis *et al*. 2014), and tested the hypothesis that the amplitude of whisker responses would be modulated according to the phase of the movie. We found that this was indeed the case, with records of individual single units showing systematic variations in whisker response across multiple presentations of the movie (Fig. 8 *C*). To quantify this effect, for individual neurons we calculated the Pearson's correlation coefficient of whisker responses across repeated presentations of this video, and repeated this analysis with whisker responses shuffled randomly across the movie phase for each repeat. Shuffling of responses resulted in a significant reduction in correlation, revealing a modulation of whisker responses according to the phase of the movie (paired two‐tailed *t* test of shuffled and unshuffled responses: *P* < 0.0001; Fig. 8 *D*).

Finally, we sought to relate the modulatory effect of the natural movie to the response of VPM units to artificial drifting gratings. Naturalistic visual stimuli are inherently complex, and often contain multiple concurrent changes in the visual scene. Nevertheless, we were able to identify particular epochs of the naturalistic stimulus during which the dominant direction of visual flow was either naso‐temporal (i.e. in the same direction as whisker deflections) or temporo‐nasal (opposite direction to whisker deflection). We expected variations in whisker responses during visual motion to be strongest for those neurons tuned to distinguish visual motion in these two directions. We therefore selected those neurons that were tuned for temporo‐nasal visual motion (based on their response to drifting grating stimuli), and examined their responses to whisker‐deflections during these two directions of visual flow. We found that responses to the naso‐temporal whisker deflection were significantly larger during epochs of temporo‐nasal visual flow (*n* = 10 temporo‐nasal tuned neurons; paired two‐tailed *t* test of amplitude during temporo‐nasal *vs*. naso‐temporal motion; *P* < 0.01; Fig. 8 *E–G*). The same was not true of neurons with no direction tuning for visual gratings (*n* = 15; paired two‐tailed *t* test *P* > 0.05; Fig. 8 *H*).

### VPM receives input from the visual cortex

**Figure 9:**
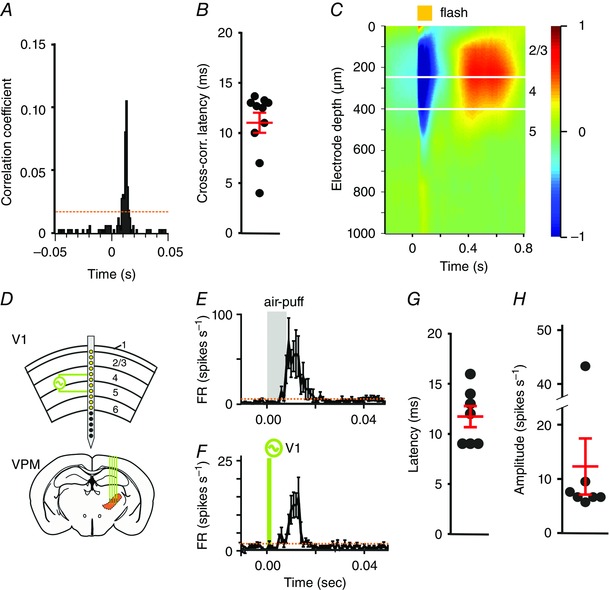
Influence of V1 on VPM physiology *A*, cross‐correlation of spiking as a function of latency (0 = time of spike in V1) for a representative pair of V1 and VPM neurons showing significant correlation (orange dashed line shows confidence interval based on 2× standard deviations of baseline). *B*, scatter of delay between firing in V1 *vs*. VPM for all V1 and VPM pairs showing significant correlation. Red lines indicate mean ± SEM. *C*, current source density (normalized CSD; −1 to 1) analysis was used to define depth from cortical surface of recording sites of a 1 × 16 probe inserted into V1. A 100 ms flash (time 0; indicated with yellow bar) evoked a laminar response profile, with a current sink in a deep layer (blue) spreading more superficially, followed by a current source (red); based upon previous reports we define these events as centred on layer 4 and 2/3, respectively (Niell & Stryker, 2008). This analysis was used to identify electrodes spanning layers 4 and 5 for electrical stimulation. *D*, cartoon depicting location of recording/stimulating electrode sites in representative placement of 1 × 16 recording probe relative to cortical layers (figures to right) derived from data in *C*, and recording probe inserted into VPM. Green lines show electrode sites spanning layers 4 and 5 used for electrical stimulation. *E* and *F*, responses of a representative unit in VPM are shown in response to 100 presentations of a 1 Hz 10 ms whisker stimulus (*E*) and to 100 presentations of a 1 Hz 100 μs current reversal in V1 (*F*). Data show mean ± SEM. Orange dashed lines show confidence interval based on 2× standard deviations of baseline firing rate. *G* and *H*, scatter of response latency (*G*) and amplitude (*H*) of neurons in VPM (7/50 whisker responsive neurons from two mice) responding to V1 electrical stimulation. Red lines indicate mean ± SEM. [Colour figure can be viewed at wileyonlinelibrary.com]

The VPM lacks direct retinal input (Morin & Studholme, 2014), raising the question of how visual signals reach it. The Allen brain atlas reports a very sparse direct projection from V1 to VPM (Experiment‐113887162. 2014). This led us to examine the possibility that visual information reaches the VPM via the visual cortex. We first addressed this by paired recordings in V1 and VPM, which would allow us to look for correlated firing and also examine the effect of silencing V1 on VPM activity. Using cross‐correlations in spontaneous spiking activity for pairs of neurons recorded simultaneously in VPM and V1 (221 pairs from four mice; Fig. 9 *A*), we identified nine pairs that exceeded our threshold for significant correlation (see Methods). In all cases firing in V1 preceded that in VPM, with a lag of 10.4 ± 1.0 ms (Fig. 9 *B*; mean ± SEM).

If VPM units are indeed downstream of V1, then we should be able to excite them using stimulating electrodes in the visual cortex. We tested this prediction and found that a 100 μs current applied to V1 induced spiking in a population of VPM neurons that were also responsive to whisker stimulation (Fig. 9 *C–H*; mean ± SEM response latency = 11.7 ± 1.9; amplitude = 12.3 ± 5.1 spikes s^–1^). The VPM response was apparent when electrical stimulation was focused on deeper layers of V1, around layer 4/5 (Fig. 9 *C*), while more superficial stimulation positions failed to evoke a response (data not shown). We also applied the GABA_A_ agonist muscimol to V1, which predictably inhibited baseline and visually evoked activity in V1. In the VPM, baseline activity was unaffected by this treatment (mean ± SEM baseline FR before and after muscimol = 3.46 ± 0.70 and 3.26 ± 0.72, respectively; paired two‐tailed *t* test, *P = *0.37), but responses to light pulses and visual gratings were abolished (mean ± SEM evoked change in FR before and after muscimol = 1.1 ± 0.29 and 0.25 ± 0.18, respectively; paired two‐tailed t test, *P = *0.004; mean ± SEM DS index before and after = 0.67 ± 0.03 and 0.21 ± 0.03, respectively; paired two‐tailed *t* test < 0.001).

**Figure 10:**
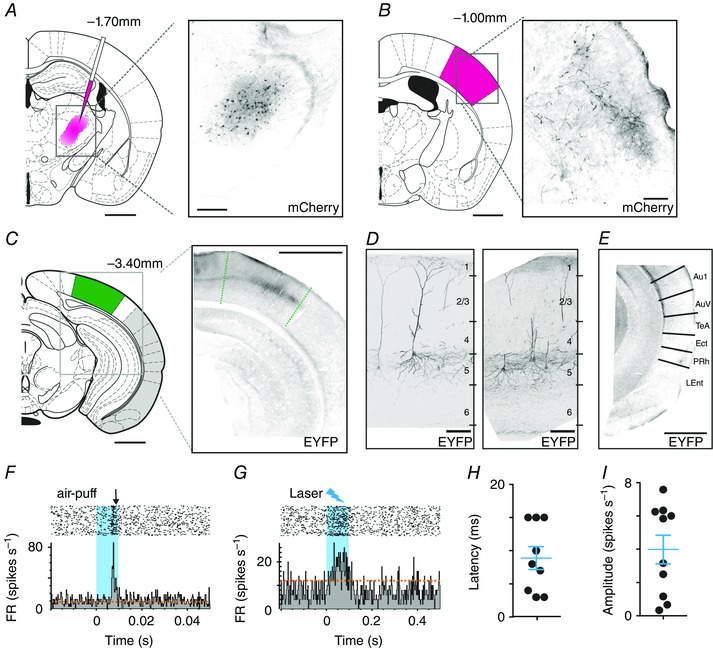
Mapping the connectivity of V1 and VPM *A*, viral tracing of connections to VPM. Left panel shows schematic depiction of injection site of AAV‐EF1a‐mCherry‐IRES‐WGA‐Cre virus (pink). Right panel shows representative coronal section of VPM, showing associated mCherry expression (black). Scale bars = 1 mm and 250 μm. *B*, left panel: schematic depiction of S1 within full coronal section. Right panel: mCherry‐positive fibres (black) of primary transfected neurons in a representative coronal section of S1 (scale bars = 1 mm). *C*, left panel: schematic depiction of V1 within full coronal section. Right panel: EYFP‐expressing neurons (black) in a representative coronal section of V1 (scale bars = 1 mm). *D*, expanded view of EYFP‐expressing cells (black) in coronal section of V1; figures to right represent approximate location of cortical layers. No mCherry expression was found in V1 (channel not shown). Scale bars = 100 μm. *E*, EYFP expression in other cortical regions (regions highlighted in grey in *C*). No EYFP signal (shown in black) was found in these regions (using equivalent exposure and gain). Abbreviations: Au1 – primary auditory cortex; AuV – secondary auditory cortex, ventral region; TeA – temporal association cortex; Ect – ectorhinal cortex; PRh – perirhinal cortex; LEnt – lateral entorhinal cortex . Scale bar = 1 mm. *F*, responses of a representative VPM single unit to repeated whisker stimulation (100 repeats 1 Hz 10 ms airpuff; blue bar indicates stimulus presentation). Upper panels show raster plots to individual stimulus repeats, and lower panels associated mean PSTH (orange dashed lines depict 2× standard deviations above baseline firing). *G*, responses of the same VPM unit shown in *F* to optogenetic activation of neurons in V1 (1 Hz presentation of 100 ms blue laser stimulus; blue bar indicates stimulus presentation). Upper panels show raster plots to individual stimulus repeats, and lower panels associated mean PSTH (orange dashed lines depicts 2× standard deviations above baseline firing). *H* and *I*, mean latency (*H*) and amplitude (*I*) of laser‐evoked firing in nine VPM units (*n = *2 mice) responding to optogenetic stimulation of V1. [Colour figure can be viewed at wileyonlinelibrary.com]

These physiological analyses are thus consistent with a functional connection from V1 to VPM. An obvious potential substrate for this connection is the direct V1 to VPM projection reported in the Allen brain atlas (Experiment‐113887162. 2014). However, this projection is limited, and is hard to reconcile with the extent of visual responses in the VPM. An alternative possibility is that a multi‐synaptic pathway(s) accounts for much of the functional connection between V1 and VPM. To allow for this possibility, we next injected a virus driving WGA‐cre expression (AAV‐EF1a‐mCherry‐IRES‐WGA‐Cre; Gradinaru *et al*. 2010) into the VPM of Ai32 mice [homozygous for the Rosa‐CAG‐LSL‐ChR2(H134R)‐EYFP‐WPRE conditional allele]. In these animals, transfected cells can be identified by their expression of mCherry. Accordingly, mCherry‐positive cell bodies were identifiable in the VPM of our injected animals (Fig. 10 *A*). The WGA‐cre recombinase expressed in these cells can then pass trans‐synaptically in both retrograde and anterograde directions to induce EYFP expression in connected neurons. As predicted given this mode of action, we found EYFP‐labelled cell bodies in the S1 region of somatosensory cortex (the major target of VPM projection neurons; not shown). More pertinent for this study, EYFP‐positive neurons were also found in V1 (Fig. 10 *C*, *D*). These labelled cells had a pyramidal morphology, with cell bodies in layer 5. EYFP was restricted to few areas of the cortex in these experiments (see Fig. 10 *E* for representative images from other cortical regions), giving confidence in the specificity of the connection we identify between V1 and VPM.

The presence of EYFP in these experiments provides evidence that neurons in V1 are connected to VPM, but does not reveal the direction of information flow in this circuit. It is notable, however, that mCherry fibres that were numerous in S1 (Fig. 10 *B*) were absent in V1, indicating that there is no direct VPM to V1 projection. In view of these data and the electrophysiological evidence summarized above, we hypothesized that the EYFP‐expressing neurons in V1 were upstream of the VPM. As cre‐recombinase activity in Ai32 mice induces ChannelRhodopsin (as well as EYFP) expression, we were able to test this possibility by optogenetic stimulation of labelled cells in V1. We found that laser stimulation in V1 induced spiking in whisker responsive neurons in the VPM (9/89 single units from two mice responding to laser; Fig. 10 *F–I*), with a response latency consistent with our unfocused electrical stimulation of V1 (mean ± SEM: 8.9 ± 1.7 ms).

## Discussion

Our serendipitous observation of light sensitivity in the VPM has led us to define a new interaction between vibrissal and visual systems in the mouse. By applying a variety of natural and artificial visual stimuli, we show that visual stimuli can influence the firing of VPM neurons and that at least one type of visual signal (optic flow) provides dynamic modulation of whisker response amplitude. In addition to describing an interaction between two of the best‐studied rodent sensory systems, our work provides an unusual example of cross‐modal interaction occurring at the level of the primary sensory thalamus.

We used several complementary approaches to isolate neurons in the VPM, giving us confidence in our conclusion that cross‐modal (visual and vibrissal) responses are found within this nucleus. Using multi‐channel recording probes that span broad regions of the thalamus (plus *post hoc* reconstruction of those recording sites), we consistently localized light/vibrissal responsive units to the VPM; these cross‐modal responses were absent when the electrode was lowered or raised to regions immediately above or below the VPM (data not shown). Using a single‐channel approach, we were able to localize and label single neurons to the VPM that responded to both visual and vibrissal responses. Functional evidence further supports these conclusions, such that visual/whisker responsive neurons we assign to the VPM show the frequency‐dependent changes in whisker responses typical of VPM, but not the neighbouring POm.

The presence of cross‐modal responses in the VPM is a surprising result, especially given the experimental attention this nucleus has received in the past. However, the unusual characteristics of visual signals in this nucleus perhaps explain why such responses would be easily missed in standard experimental protocols. When presented with a bright, full field stimulus, unlike the precise and repeatable influence of visual responses in retinorecipient brain regions, light‐responsive neurons in the VPM show a long latency to reach a maximum response, building up over several seconds of presentation. Likewise, drifting gratings did not induce modulations in firing at the frequency expected for cells tracking the grating phase, as is commonly the case in visual areas. Instead, it was their tonic firing rate that was affected.

The VPM is not a retinorecipient region, and several aspects of our data lead us to conclude that visual information reaches it via the primary visual cortex. In the first place, VPM responses to both simple light steps and drifting gratings are inhibited by muscimol application to V1. Conversely, either electrical or optogenetic stimulation of V1 drives spiking in VPM. Finally, simultaneous recordings in V1 and VPM reveal coordinated firing of neurons in these two brain regions. These physiological analyses are thus consistent with a functional connection from V1 to VPM. Nevertheless, the latency between V1 and VPM spikes revealed in our paired recording and optical/electrical stimulation experiments (∼11 ms) is most consistent with this signal travelling more than one synapse. This does not preclude the possibility that these responses originate with the reported sparse direct projection from V1 to VPM (Experiment‐113887162. 2014). However, in that case connections within the VPM would be required to transfer visual information to most of the cells from which we record. Alternatively, established V1 projections to thalamus, midbrain, striatum or higher visual areas within the cortex (see Wang & Burkhalter 2007; Oh *et al*. 2014) allow plenty of opportunity for visual signals to reach individual VPM neurons via a more circuitous route: for example, via known projections from V1 to S1 (Sieben *et al*. 2013; Stehberg *et al*. 2014), and then from cortical layer V to VPM. Alternatively, cortico‐thalamic inputs from V1 to other thalamic regions (e.g. LP, LGN, POm) might also relay visual signals to VPM. Establishing the precise nature of this route is an exciting area for future study.

The description of light responses in the VPM raises the question of what visual features neurons in this brain region are responsive to. The work presented here does not represent a comprehensive answer to this question. We do, however, find that ∼50% of light‐responsive VPM neurons were particularly excited by stimuli recreating visual motion. Thus, while no VPM units tracked the alternations in local radiance provided by drifting gratings, many showed changes in baseline firing according to the direction in which the gratings were moving. A similar response could be evoked by moving squares, but only if multiple squares moved at once and, especially, if they moved in the same direction. Such stimuli recreate ‘optic‐flow’, which is commonly encountered when there is relative motion between the observer (or eye) and the visual scene.

Optic flow is an established method of detecting self‐motion. In mice, measuring optic flow can contribute to cortical representations of running speed (Saleem *et al*. 2013), and is an established method of tracking eye/head movements in oculomotor control (Oyster & Barlow, 1967; Oyster *et al*. 1972; Simpson *et al*. 1988; Soodak & Simpson, 1988). It is also known that other measures of self‐motion, including locomotion speed and vestibular signals, influence activity in primary sensory pathways [including in S1 (Sofroniew *et al*. 2015), dLGN (Erisken *et al*. 2014) and LP (Roth *et al*. 2016)]. Our experiments, in which we have presented visual stimuli to an immobilized mouse, recreate the visual sensation of motion without any of the proprioceptive/vestibular inputs that might also be associated with movement. This manipulation has revealed a new use for visual signals in encoding self‐motion.

We find that both natural and artificial representations of optic flow alter the representation of passive whisker movements in the VPM, so that whisker responses are enhanced in the presence of visual motion in the *opposite* direction; during natural movement, passive whisker deflections should mostly occur in the *same* direction as visual motion, as whiskers encounter objects or are deflected by air‐flow as the mouse's head moves through the environment. The interaction we describe might therefore accentuate less expected deflections in the opposite direction, and represent an example of how sensory systems deal with inputs generated by self‐motion (reafference; von Holst & Mittelstaedt, 1950). It would thus provide a simple mechanism for establishing a saliency hierarchy for vibrissal signals before they reach the cortex.

## Additional information

### Competing interests

The authors declare no competing financial interests.

### Funding

This work was funded by the BBSRC (BB/I007296/1 to R.J.L.) and ERC (268970 to R.J.L.).

### Author contributions

A.E.A., C.A.P., T.M.B. and R.J.L. designed the experiments, A.E.A. and C.A.P. collected and analysed the data, and A.E.A., T.M.B. and R.J.L. wrote the manuscript. All authors approved the final version of the manuscript, agree to be accountable for all aspects of the work and all persons designated as authors qualify for authorship. All experiments were performed at the University of Manchester, UK.
